# Resource-dependent investment in male sexual traits in a viviparous fish

**DOI:** 10.1093/beheco/arac060

**Published:** 2022-06-23

**Authors:** Erika Fernlund Isaksson, Charel Reuland, Ariel F Kahrl, Alessandro Devigili, John L Fitzpatrick

**Affiliations:** Department of Zoology, Stockholm University, Svante Arrhenius väg 18B, 10691 Stockholm, Sweden; Department of Zoology, Stockholm University, Svante Arrhenius väg 18B, 10691 Stockholm, Sweden; Department of Zoology, Stockholm University, Svante Arrhenius väg 18B, 10691 Stockholm, Sweden; Department of Zoology, Stockholm University, Svante Arrhenius väg 18B, 10691 Stockholm, Sweden; Department of Biology, University of Padova, Via Ugo Bassi 58B, 35131 Padova, Italy; Department of Zoology, Stockholm University, Svante Arrhenius väg 18B, 10691 Stockholm, Sweden

**Keywords:** condition dependence, intra-sexual competition, mate choice, resource manipulation, sexual selection

## Abstract

Exaggerated and conspicuous sexually selected traits are often costly to produce and maintain. Costly traits are expected to show resource-dependent expression, since limited resources prevent animals from investing maximally in multiple traits simultaneously. However, there may be critical periods during an individual’s life where the expression of traits is altered if resources are limited. Moreover, costly sexual traits may arise from sexual selection acting both before (pre-copulatory) and after mating (post-copulatory). Gaining a robust understanding of resource-dependent trait expression therefore requires an approach that examines both episodes of sexual selection after resource limitation during different times in an individual’s life. Yet few studies have taken such an approach. Here, we examine how resource restriction influences a set of pre- and post-copulatory traits in male pygmy halfbeaks (*Dermogenys collettei*), which invest in sexual ornaments and routinely engage in male–male contests and sperm competition. Critically, we examined responses in males when resources were restricted during development and after reaching sexual maturity. Both pre- and post-copulatory traits are resource-dependent in male halfbeaks. Body size, beak size, courtship behavior, and testes size were reduced by diet restriction, while, unexpectedly, the restricted-diet group had a larger area of red color on the beak and fins after diet treatment. These patterns were generally consistent when resources were restricted during development and after reaching sexual maturity. The study reinforces the role of resource acquisition in maintaining variation among sexual traits.

## Teaser

Hide Does going on a diet make animals worse at reproducing? Evolutionary theory suggests that reproductive success often depends on how good animal are in finding food and converting this energy into finding and attracting mates. We tested this idea in a small freshwater fish and show that males with access to less food are worse at courting females, have shorter jaws, and produce fewer sperm.

## INTRODUCTION

Sexual selection is expected to promote the evolution of traits that increase success in intra-sexual competition and inter-sexual mate choice (Darwin 1871; Andersson 1994). Such sexually selected traits can be costly to produce and maintain and their expression can therefore depend on the condition of the bearer (Johnstone et al. 2009). Condition is typically defined as the pool of resources an individual has acquired for investment in traits that increase fitness (Rowe and Houle 1996) and is often closely coupled with resource availability (Kotiaho 2000; Cotton et al. 2004; Macartney et al. 2019). Thus, resource-dependent expression of costly sexual traits is expected to serve as an honest signal of individual quality (Zahavi 1975). However, as resources are often limited in natural habitats, theory predicts that investing in one trait reduces the pool of resources available for allocating to other traits (Rowe and Houle 1996; Simmons et al. 2017). Resource allocation patterns will therefore influence how traits are expressed (Simmons et al. 2017). Moreover, sexual selection can occur before (pre-copulatory) and after (post-copulatory) mating (Andersson 1994; Birkhead and Pizzari 2002) and acts on multiple traits that influence individual fitness simultaneously (Evans and Garcia-Gonzalez 2016). Resource-dependent trait expression may therefore vary among sexual traits, and traits that produce larger marginal fitness gains after increased investment are more likely to exhibit resource-dependence (Fox et al. 2019). Therefore, attempting to explain investment in sexual traits requires an approach that assesses numerous traits that function in both intra- and inter-sexual selection, which influence fitness both before and after mating.

A powerful method to examine the resource dependence of sexual traits is by experimentally manipulating resource availability. Experimentally restricting available resources reduces or alters sexual behaviors and the phenotypic expression of a range of sexual traits in numerous animal groups (Cotton et al. 2004, 2006; Macartney et al. 2019). For example, pre-copulatory courtship behaviors, such as acoustic signaling and display rates, are commonly reduced after resource restrictions (e.g., Cotton et al. 2004; Immonen et al. 2009; Devigili et al. 2013; Rahman et al. 2013; Rahman et al. 2014; Evans et al. 2015; Kaldun and Otti 2016; Cattelan et al. 2019; Fox et al. 2019). Sexual weapons used in intra-sexual competition are also sensitive to resource restrictions (e.g., mandible size in armed beetles, *Gnatocerus cornutus*, Katsuki et al. 2012). Similarly, sexual ornaments displayed by courters (e.g., bright colorations), and used by choosers to assess courter quality, are reduced after experimental restriction of resources (Cotton et al. 2004). The expression of sexual ornaments can decrease after even relatively short durations of food restriction (e.g., ~1 month; Devigili et al. 2013). Yet, while the available experimental evidence suggests broad support for resource-dependent expression of pre-copulatory sexual traits (Cotton et al. 2004), there remain conspicuous examples where different aspects of sexual behaviors and ornaments are not affected by resource restriction (e.g., Hill 1993; Perez-Rodriguez and Vinuela 2008; Devigili et al. 2013; Rahman et al. 2013).

Sexual selection can also favor the evolution of exaggerated post-copulatory traits linked with fertilization success during sperm competition, such as sperm number and quality (Pitnick et al. 2009; Simmons and Fitzpatrick 2012; Fitzpatrick and Lüpold 2014; Lüpold et al. 2016, 2020). As ejaculate traits can be costly to produce and maintain (Olsson et al. 1997; Lüpold et al. 2016), sperm number and quality are also expected to exhibit resource-dependence. In a recent meta-analysis assessing 50 species ranging from arthropods to mammals, Macartney et al. (2019) reported that testes size and seminal fluid quantity showed the strongest reduction from resource restriction across animal groups. Sperm quantity and motility were also negatively impacted by resource restriction, albeit to a weaker degree (Macartney et al. 2019). Sperm length and the percentage of normally formed sperm in the ejaculate (i.e., sperm normality) were not influenced by resource restriction (Macartney et al. 2019). However, patterns of resource-dependence among ejaculate traits differed among fishes, mammals, and arthropods, the three most well represented taxa in Macartney et al.’s (2019) analysis. Interestingly, in fishes overall, sperm length and morphological normality are more negatively affected by resource restriction than sperm quantity and motility (with some exceptions, e.g., Gasparini et al. 2013), a finding that contrasted the pattern detected in the overall analysis across 50 animal species, as well as the result in arthropods and mammals (Macartney et al. 2019). These taxa-specific responses suggest that resource-dependent expression of post-copulatory traits in fishes may differ from other animal groups. Moreover, since the ejaculate and their subcomponents share the same purpose of fertilizing eggs, these diverse patterns of resource-dependence among ejaculate traits highlight the importance of assaying a wide range of traits.

While costly sexual traits are generally expected to exhibit resource-dependence, such effects may fundamentally depend on when resources are limited during an individual’s lifetime. Specifically, responses to resource restriction may depend on an individual’s developmental stage, as limiting resource during development versus after reaching sexual maturity may influence trait expression differently (e.g., Katsuki et al. 2012; Macartney et al. 2019). Macartney and colleagues’ meta-analysis revealed a slightly stronger response generally when resources were restricted during development than after sexual maturity (Macartney et al. 2019). Interestingly, fish seem to divert from this general pattern by responding stronger when resource restriction occurred after reaching sexual maturity (Macartney et al. 2019). The influence of resource availability during specific life stages will also be influenced by the plasticity of the traits measured. For example, spermatogenesis occurs throughout adulthood in many animals and is therefore expected to be influenced by resource restriction at any point after reaching sexual maturity (Macartney et al. 2019). Structural traits, on the other hand, may rely more on investment during development rather than at maturity, and should therefore be less influenced by limited resources late in life than during development (Katsuki et al. 2012). Thus, gaining a clear understanding of resource-dependent expression of sexual traits requires assessing the impact of resource restriction at different developmental stages.

Here, we experimentally examine resource dependence in a suite of pre- and post-copulatory traits and compare the effects of resource restriction at different developmental stages in the pygmy halfbeak (*Dermogenys collettei*). Pygmy halfbeaks are small, viviparous, internally fertilizing, and promiscuous fish from Southeast Asia. Halfbeaks are characterized by an elongated lower jaw (henceforth called “beak”, see Supplemental Fig. 1). Beaks are used in intra-sexual competition, where halfbeaks bite each other or lock their beaks together (“beak-locking”, or “wrestling”; Berten and Greven 1991; Greven 2006, 2010), which may be important in determining the outcome of escalated aggressive encounters. Male halfbeaks perform a series of well-characterized, often time-consuming, courtship displays (Greven 2010) and a recent study from natural populations showed that males spend on average one third of their time courting females (Devigili et al. 2021). Halfbeaks are sexually dimorphic in their coloration, with males investing in a greater amount of yellow, red, and black coloration on their fins and bodies than females (Reuland et al. 2020). Female halfbeaks pay attention to the red coloration on the body of males when making mate choice decisions in dichotomous choice trials (Reuland et al. 2020; McNeil et al. 2021). The direction of female preference is influenced by female mating status (Reuland et al. 2020) and the magnitude of the differences in red coloration between the males (McNeil et al. 2021). If red coloration in males is resource-dependent, it may explain some of the observed variation in female preference. Additionally, male halfbeaks invest substantially in testicular tissue (~3–6% of their body weight (Ogden et al. 2020; see Results), compared with a mean (±SE) of 1.66 ± 0.24% (range: 0.05–9.10%) from a sample of 91 fish species (Baker et al. 2020)). Females commonly mate with multiple males and store sperm across their brood cycle (Ogden et al. 2020). Thus, sperm competition appears to be a salient evolutionary force in these fish.

The rare presence in the same species of sexual ornamentation, elaborate courtship behaviors, a possible weapon for intra-sexual competition (beak), and large post-copulatory investment makes the halfbeak an ideal model to examine resource dependence of a range of sexually selected traits. We capitalize on these attributes to contrast patterns of resource dependence across sexual traits after resource restriction during either juvenile development or after reaching sexual maturity in male halfbeaks. We then assess whether resource restriction in males influences the outcome of male–male competitive interactions and female mate preferences in halfbeaks. We predict that overall, 1) sexual traits are costly to produce and maintain and will therefore show reduced expression after resource restriction, 2) resource-dependent trait expression will be stronger when resource restriction occurs during development than when applied after males are sexually mature, in particular for structural traits (such as beak length), 3) females will prefer males with more exaggerated expression of resource-dependent sexual traits, and 4) males on resource-restricted treatments will be competitively inferior during male–male competition.

## METHODS

### Study population and housing conditions

Experimental fish were generated from F1 and F2 descendants of wild-caught halfbeaks collected from Sungai Tebrau, Malaysia, that were bred in an aquarium facility at Stockholm University, Sweden. The adult fish, which live for up to ~3 years in captivity (personal observation), used to produce the experimental subjects were kept in mixed-sex groups in 160 L aquaria containing ~2 cm of gravel, aeration, and live and artificial plants. Fish were maintained on a 12:12 h light:dark cycle at ~26 °C. Offspring were collected either from mixed-sex groups or individually housed gravid females (females were separated from offspring shortly after birth to prevent infanticide) and then used in one of the two diet manipulation experiments described below. The two experiments were performed consecutively over the course of 6 months in 2019 and 8 months in 2020, respectively. Experiments were approved by Stockholm Animal Research Ethical Board (permit number 2393-2018 and 3967-2020).

#### Experiment 1: adult diet experiment

In this experiment, halfbeaks were reared on different experimental diet treatments after reaching sexual maturity (henceforth called the *adult diet experiment*). Offspring were reared until sexual maturity under standard laboratory conditions (see above). After birth, up to five offspring (~3–5 mm in length) were housed together in 4 L tanks for up to 2 months, during which they are fed exclusively live *Artemia salina* nauplii (henceforth referred to as artemia) twice per day. 20–30 fry from different 4 L tanks that were born within the same calendar month were then moved to 72 L tanks until the onset of sexual maturity. During this period of development, juveniles were fed ad libitum with a mixture of ground flake food and freeze-dried artemia twice per day, 6 days per week. In addition, previously frozen and thawed *Drosophila melanogaster* and live artemia were fed to fish once and 3–4 times per week, respectively. At the onset of sexual maturity (~2 months of age), males were identified by the developing of the modified anal fin (the andropodium) used for copulation (Meisner and Burns 1997). After identification, males were removed from the 72 L tanks and reared in male-only tanks (55 or 160 L, 15–30 males per tank) until ~3–4.5 months of age (ensuring sexual maturity) when they were allocated to an experimental diet treatment.

To experimentally manipulate adult diets, sexually mature males (*n* = 70) were taken from male-only tanks at age ~3–4.5 months and placed in individual 4 L tanks (25 × 16 × 12 cm) containing ~2 cm of gravel, four floating plastic plants, 2–3 snails, and constant aeration. A floating circular feeding arena (a hollow plastic tube) was placed in the center of the tank. The feeding arena was used to ensure that fish could easily find and access the food. Fish were allocated to one of two experimental diet treatments: a high or restricted quantity diet. There was no difference in body size between groups prior to diet treatment (linear model; *F* = 0.11, df = 1, *P* = 0.74). Of the original sample size of 70 males, 4 died during diet treatment (2 from high diet and 2 from restricted diet treatment), reducing the final sample size to 66 males (33 in each treatment). In the *adult high-diet* treatment (*n* = 33), each week males were fed six fruit flies (*D. melanogaster*) per day (3 in the morning, 3 in the afternoon) for 5 days and 3 flies per day for 2 days (total per week: 36 flies). In the *adult restricted-diet* treatment (*n* = 33), each week males were fed one fruit fly per day for 5 days and 2 flies per day for 2 days (total per week: 9 flies). We used only fruit flies to manipulate adult diets because they present a simple way to control food quantity. Males were maintained on these diet treatments for 28–31 days. Note that assays were initiated around day 21 of the diet treatment and continued until termination at day 28–31. In all tanks, approximately 10% of the water was changed three times per week, using a flow through system to minimize disturbance. The adult diet experiment was performed in 4 blocks (*n* = 16–18 males per block, equalized between treatment groups).

Assays (described below) were performed in the following order, each separated by 1–3 days: female mate choice assay, courtship assay, competition assay, morphological and post-copulatory measurements (lateral pictures for body length, beak length, and coloration traits, body mass measurement, sperm assays, and testes mass measurement).

#### Experiment 2: developmental diet experiment

In this experiment, halfbeaks were reared on different experimental diets treatments until reaching sexual maturity (henceforth called the *developmental diet experiment*). New-born fish (*n* = 111 fry < 3 days old, unsexed, that is, a mix of males and females) were collected from stock tanks and placed in 4 L experimental tanks. For the first 28 days of the experiment, juveniles were kept in groups of 2–4 individuals and fed an ad libitum diet of live artemia twice per day. This initial ad libitum diet stage was done to minimize juvenile mortality, which is greatest during the first month after birth. After these initial 28 days, juveniles were placed in individual 4 L tanks containing 2 cm of gravel, plastic plants, 2–3 snails, and constant aeration. Juveniles do not eat *D. melanogaster* and were therefore maintained on a diet of live artemia. Juveniles were allocated to either a high or restricted-diet treatment. In the *developmental high-diet* treatment, fish were fed 3 ml of the solution obtained from 6 ml artemia cysts hatched in 1200 ml water solution twice per day for 6 days each week (12 × 3 ml feedings per week). Fish in the restricted-diet treatment were fed increasing amounts of food as they grew after the initial 28 days of ad libitum feeding to account for increases in fish size throughout the experiment. The same concentration of artemia was used throughout the experiment. During the first, second, and third month of the diet treatment, juveniles in the restricted-diet treatment were fed 3 ml of the artemia solution 1) once per day, 3 days per week (3 × 3 ml feedings per week), 2) once per day, 5 days per week (5 × 3 ml feedings per week), and 3) twice per day for 1 day per week and once per day for 5 days per week (7 × 3 ml feedings per week), respectively. Fish were maintained on these diet treatments for a total of 3–3.5 months, which included the last 1.5 week during which assays were performed.

Halfbeaks live in shoals and interactions with conspecifics are likely a normal part of their development (e.g., Devigili et al. 2021). Therefore, after isolation in individual rearing tanks (i.e., after the initial 28 days of group rearing), all juveniles were exposed to a conspecific once every 3 weeks (i.e., at week 3, 6, 9, 12, and 15 of diet treatment). During these exposures, one juvenile fish (the “visitor”) was placed into a transparent plastic cylinder (12 cm in diameter), which was subsequently placed inside the tank of another juvenile fish (the “host”) for 4–6 h (approximately 10 AM–3 PM). The cylinder was perforated, with ~2 mm holes placed around the cylinder, to allow visual and olfactory cues to be assessed between the visitor and host fish, while preventing direct physical interactions. All fish were fed at least 1.5 h before the exposures to minimize the risk of live artemia being left in the home tanks when the visitor was added. Each juvenile acted as the visitor or host an equal number of times over the course of the experiment. During each social exposure, juveniles were paired with a novel individual from the same experimental block (see below).

Of the initial 111 juveniles that entered the experiment, 10 died over the course of the experiment (8 pre-treatment, 1 from high-diet treatment, 1 from low-diet treatment), reducing the sample size to 101 fish (mix of males and females). The experiment was performed in three blocks, where fry born within 2 weeks were pooled into a single block and assayed together. Each of the blocks contained between 32 and 36 juveniles, with diet treatments equalized within each block. When focusing only on males, this translated into 18–25 males per block. The final sample size in the developmental diet experiment was 50 males (*n* = 27 in the restricted diet treatment and *n* = 23 in the high diet treatment). At 4–4.5 months of age, the assays were initiated. Note that since all assays take approximately 10 days to complete per block, diet treatments continued during this time. The order of the assays was the same as in the adult diet experiment (see above) with the exception that a lateral picture (see below) was captured the day before female mate choice in order to produce the size-matched pairs of males.

### Male body size and external morphology

Body size and external morphology were quantified for males in both experiments using photographs. The lateral side of each male was photographed using a digital camera (Canon 800D with macro lens EF-S 60 mm) inside a photo chamber (30 × 20 × 20 cm) under standard light conditions. Each image included a scale bar. From these images, body length was measured in ImageJ (v1.52i; Schneider et al. 2012) from the anterior part of the eye to the caudal peduncle (Supplemental Fig. 1). This measure of body length excludes the jaw, which is variable among males. We hypothesized that the length of the beak, which may be important in both male–male competition and female mate choice (Berten and Greven 1991; Greven 2006), could exhibit resource dependence and this could be independent of the rest of the body. We therefore measured the beak length separately from the rest of the body, from the anterior tip of the beak to the anterior part of the eye (Supplemental Fig. 1). Additionally, we measured the area of the yellow and red coloration on the caudal, dorsal, and anal fins, black coloration on the dorsal fin, and red and black coloration on the beak using the polygon selections tool in ImageJ (v1.52p) by tracing around the colored areas of the respective body parts. For the analysis, we included only normally developed beaks (i.e., straight), reducing the sample size to 98 males (adult diet experiment: high *n* = 30; restricted *n* = 23; juvenile diet experiment: high *n* = 22, restricted *n* = 23).

To examine coloration on the fins and beak, we used a principal component analysis (PCA) to reduce the dimensionality of the data, thereby reducing the number of statistical tests (and the associated risk of Type-II errors) and account for potential collinearity among variables. The total amount of red, yellow, and black coloration on the fins and red and black coloration on the beak were entered into a PCA. The PCA returned 2 PCs (henceforth coloration PC1 and PC2) with standard deviations ≥ 1, which together accounted for 56% of the total variance and were used for further analysis (Supplementary Table 1). Loading values of each PC are considered to contribute to that PC when the loading values are 70% of the variable with the highest loading. Coloration PC1 was primarily loaded by yellow fin coloration and black beak coloration, while coloration PC2 was primarily loaded by red beak coloration and the total red coloration on the fins (Supplementary Table 1).

Body mass was measured by placing fish individually in a small dish containing water on a previously tared balance (Mettler Toledo XS105). This procedure was repeated for two independent mass measurements per individual and an average of the two measurements was used for analysis. Body length and mass were measured at the start and end of the diet treatments in the adult diet experiment, while measures were only taken at the end of the diet treatments in the developmental diet experiment. We estimate body condition using Fulton’s condition factor (*K* = (body mass/length^3^) * 100), a common proxy of condition in fish (Nash et al. 2006).

### Male courtship behaviors

Male courtship behaviors were recorded using a free-swimming assay where one focal male could interact with one female. Individual males from each of the adult and juvenile diet treatment groups were added to an experimental tank (dimensions 40 × 24 × 30 cm) filled to a water depth of 15 cm, containing two plastic plants and ~1 cm of gravel. A sexually mature (i.e., >4 months old) virgin female randomly chosen from a female-only stock tank was then added to the experimental tank. The male and female were separated by a transparent plastic divider and left undisturbed for 1 h, allowing the fish to habituate to the experimental tanks. After the habituation period, the transparent divider was lifted using a pulley system, allowing the male and female to interact. During a 20 min observation period, we recorded a number of behaviors. As discrete behaviors, we recorded the number of male *circling*, where the male swims around the female’s head in a semicircle, which may be an initial mate assessment behavior, and *matings*, a rapid movement (~40–80 ms) where the male twists his body to attempt to insert the andropodium into the females genital pore (Greven 2010; note that it is impossible to distinguish between successful or unsuccessful mating attempts solely from behavioral observations). As continuous behaviors, we recorded the time a male was observed *swimming under* the female while positioned ventrally and posteriorly with his head directly below the female’s genital pore, *nipping*, where the male is swimming under the female but now moves his upper and lower beaks, rapidly clapping the upper and lower beak together, and *checking* at genital pore, where the male makes physical contact with the female’s genital pore using his upper jaw. Behaviors were recorded in real-time by one experimenter (E.F.I.). Fish were fed ~2 h prior to all courtship assays. Trials for all courtship assays were performed and analyzed blind to the treatment group by giving the males a unique identifier code.

We summed the behaviors measured as counts (circling and mating) to generate a *total number of sexual behaviors* index. Similarly, all behaviors measured as durations (time spent under female, nipping, or checking at the genital pore) were summed together to generate a *total duration of sexual behaviors* index. After summing the behaviors, these indices were divided by the amount of time that the focal male was aware of the female, which was obtained by subtracting the time from the start of the trial until the male and female first interacted from the total time of the trail. Courtship trials were aborted if the male or female showed signs of stress (such as repeatedly swimming against the glass; *n* = 6) or if either fish acted aggressively towards the other (usually the female towards the male; *n* = 4). Thus, the final sample size for the courtship assays was 106 trials (adult diet experiment, high *n* = 30; restricted *n* = 30; developmental diet experiment, high *n* = 20, restricted *n* = 26).

### Male–male competition behaviors

Competition behaviors were video-recorded in a free-swimming assay where one male from the high-diet treatment and one from the restricted-diet treatment were allowed to interact. To examine the effect of resource restriction on competitive interactions removing body size-mediated effects, males in each trial were size matched prior to the assay with a maximum difference of 3 mm in length allowed between the males in the pair. On average, the difference in length within the pair was 0.44 ± 0.08 mm (mean ± SE) in the adult diet experiment and 1.43 ± 0.16 mm in the developmental diet experiment. In two blocks of the developmental diet experiment, there were two more fish in the juvenile restricted-diet treatment than in the juvenile high-diet treatment. Therefore, these four fish created pairs with two males of the same treatment. These pairs were treated the same as regular pairs (i.e., went through the competition assay and were recorded), in order for all fish to have the same experience for subsequent assays. These two recordings were not included in the analysis (see below).

The competition assays were recorded from above with a camera (Point Grey Grasshopper 3 4.1 megapixel camera with Fujinon CF25HA-1 lens) placed 1.5 m directly above a circular experimental tank (50 cm in diameter, containing 4 cm of water). Each male in a pair was first placed in a separate opaque cylinder (15 cm in diameter) inside the experimental tank and allowed to acclimate for 15 min without physical or visual contact with the other male. After the acclimation period, the recording was initiated, and the cylinders were lifted by hand. The recording proceeded for 20 min. The video recordings were subsequently scored blind by one experimenter (E.F.I.). In each trial, a randomly chosen focal individual was selected and their behaviors were recorded by keeping track of the focal fish frame by frame (we refer to the other fish in the pair as the “rival fish”). We scored the number of displacements (where one fish swims up to the other from the side or front, causing the other to divert), the number of lunges (one fish charges quickly at the other fish and makes physical contact with its beak somewhere on the body of the other fish), and the duration of swimming behind (the fish is swimming up to five body lengths behind the other, facing in the same direction, measured in seconds). All behaviors were scored from the perspective of the focal fish, recording the number and duration of behavior *performed* by the focal fish and directed at the rival fish or the number and duration of behaviors that were *experienced* by the focal fish (i.e., performed by the rival fish). Lunges and displacements were considered aggressive behaviors. In total, 58 trials were recorded (33 in the adult diet experiment and 25 in the developmental diet experiment). Scoring of behaviors stopped before the end of the recording (i.e., reducing the observation time) if the observer lost track of the IDs of the experimental individuals (*n* = 10).

For analysis of competitive behaviors, we converted the behaviors *experienced* by the focal individual to *performed* by the rival, in order to have a comparable value for the males of the two treatments. Therefore, the data was analyzed per individual, rather than per recording. Since each recording is represented two times in the data, we also control for recording ID in the analysis. The behaviors measured as counts (displacements and lunges) were summed per individual. Count behaviors and the duration behavior were divided by the total trial time. Trials had to be a minimum duration of 10 min of interactions to be included in the analysis, thus excluding four shorter trials. Three recordings were excluded due to technical errors while recording. Two recordings were excluded since the males in the pairs were from the same diet treatment (see above), leading to a final sample size of 100 individuals (adult diet experiment, high *n* = 30; restricted *n* = 30; developmental diet experiment, high *n* = 20, restricted *n* = 20; from 50 recordings).

### Female preference assay

A dichotomous choice assay was used to assess whether male resource-dependent traits influence the outcome of female mate preferences. For both the adult and developmental diet experiments, one female and two males (one from each diet treatment) were placed in an experimental tank (45 × 25 × 20 cm), consisting of one main chamber (45 × 15 × 20 cm), and 3 adjacent, isolated stimulus chambers (each 15 × 10 × 20 cm, see Fig. 2 in Reuland et al. 2020). The same individuals were used in the pairs for the female mate choice assay as for the competition assay (i.e., one high and one restricted diet male). A focal virgin female between 4 and 6 months old was randomly chosen from a female-only stock tank and placed in the main chamber, and the two males were randomly placed each in either the left or right stimulus chamber. The middle stimulus chamber contained no fish but was filled with gravel and water for the purpose of spatially separating the right and left chambers. Transparent glass walls separated the main chamber and the three smaller stimulus chambers. Thus, the focal female could only use visual cues to assess the stimuli males. Opaque walls separated the three stimulus chambers to prevent visual contact between stimuli males. The entire experimental tank was surrounded by opaque dividers to reduce the potential for external factors to influence the fish’s behavior.

Before each trial, fish were given 60 min to habituate to the environment, during which time visual contact between all chambers was prevented by an opaque divider. After the habituation time, the divider between the main and stimulus chambers was lifted using a pulley system and trials were recorded for 60 min using a GoPro Hero 5 Black digital camera (GoPro, Inc., San Mateo, CA, USA) or a webcam (Logitech C920 1080P HD, Carl Zeiss Tessar) positioned 30 cm above the tank. The duration of time a female spent in the left or right association zones (an area of 5 × 15 cm in front of each stimulus chamber) was used as a measure of female preference for the male in that chamber. Association time is a commonly used proxy of mating preference, which is predictive of preference during real mating interactions (Bischoff et al. 1985; White et al. 2003; Walling et al. 2010). To quantify informed choice (or preference) we only considered association times after the choosing individual had visited the association zones of both stimulus individuals. The water in the tank was changed between trials to avoid any remaining olfactory cues from the previous trial.

In total, 55 trials were conducted. In a few cases, the female never visited the second male (*n* = 7), or visited him late into the video, resulting in less than 10 min of informed choice (*n* = 3). Two recordings from the developmental diet experiment where both of the two males in the pair were of the restricted diet treatment were removed (*n* = 2). Additionally, trials were excluded if the female or one or both males behaved in a stressed manner (*n* = 4). The final sample size consisted of 39 trials (adult diet experiment, *n* = 22; developmental diet experiment, *n* = 17).

### Sperm quality and testes size

At the end of the diet treatments, and after behavioral assays, males were euthanized in a benzocaine solution (150 µl benzocaine per 1 ml ethanol), washed in deionized water and placed on a glass slide containing ~1 ml of a 9% saline (NaCl) solution, and viewed under a dissection microscope (S9 stereo microscope, Leica Microsystems, Wetzlar, Germany). Male halfbeaks produce sperm bundles called spermatozeugmata (Grier and Collette 1987). Sperm bundles were extracted into the saline solution by applying gentle pressure with a blunt instrument to the abdomen and moving towards the posterior part of the testicular duct, the channel that transports sperm from the testes to the andropodium, a modified anal fin used to transfer sperm to females (Greven 2010). 20 µl of the sperm/saline solution was then transferred to an Eppendorf tube containing 20 µl of activation solution (Hank’s Balanced Salt Solution (HBSS), Sigma-Aldrich, United Kingdom) in a 1:1 ratio of sperm/saline to HBSS for use in subsequent analyses.

Sperm velocity, viability, and morphology were measured from separate subsets from the activated sperm/saline solution using an integrated semen analysis system (ISAS, v. 1.2.33, PROiSER R + D, Paterna, Spain). Since they are measured from different subsamples, sample size may differ between traits (see below). To assess sperm velocity, 3 µl of the sperm/saline solution was transferred to two wells of a 12-well multitest slide (MP Biomedical) coated with a 1% polyvinyl alcohol (PVA) solution and covered in a previously coated PVA cover glass to prevent cells sticking to the glass (Wilson-Leedy and Ingermann 2007). Sperm swimming parameters were characterized using computer-assisted sperm analysis (CASA) software (UB 200i Series Microscope and C13-ON camera, PROiSER R + D, Paterna, Spain). Of the total of 116 males from both experiments, the velocity subsamples of 8 males had insufficient sperm to facilitate sperm measurements, reducing the sample size to 108 males (adult diet manipulation, high *n* = 30; restricted *n* = 32; juvenile diet manipulation, high *n* = 23, restricted *n* = 23). Only velocity samples with a minimum of 20 sperm cells measured were used, and the threshold for defining static cells was predetermined at 25 µm/s for VCL. Applying these exclusion criteria reduced the final sample size to 93 males (adult diet manipulation, high *n* = 26; restricted *n* = 23; juvenile diet manipulation, high *n* = 22, restricted n = 22). From these males, sperm velocity was measured for a mean (±SE) of 102.76 ± 12.17 and 183.59 ± 15.52 sperm cells per male in the adult and juvenile diet manipulation experiments, respectively. We focus on three commonly used, colinear sperm velocity metrics obtained from CASA, including the average path velocity (VAP), straight-line velocity (VSL), and curvilinear velocity (VCL). These three sperm motility measures were reduced using a PCA. The PCA returned one PC with standard deviation ≥ 1, which accounted for more than 96% of the total variance and was used for further analysis (Supplementary Table 2). Sperm velocity PC1 was loaded by all three measures of sperm velocity (VSL, VCL, and VAP) (Supplementary Table 2).

Sperm viability, quantified as the proportion of live sperm cells in each ejaculate, was measured using a live/dead cell viability kit (VitalTest, NordicCell, Denmark). A 14 µl subsample from the sperm/saline/HBSS solution was transferred to a new Eppendorf tube and 1.6 µl of propidium iodide and 0.5 µl of acridine orange were added. After adding the dyes, 14 µl (2 samples of 7 µl each) of stained sperm solution was transferred to a microscopic slide and left for 1–2 min in darkness, after which it was placed under a fluorescent microscope, and images were captured (×200 magnification; UB 200i Series Microscope and C13-ON camera, PROiSER R + D, Paterna, Spain). Viable sperm with an intact outer membrane are labelled green by membrane-non-permeable dye (acridine orange) while dead or inviable cells with disrupted membranes are labelled red with membrane-permeable stain (propidium iodide). Sperm viability was then calculated based on a mean (±SE) of 244.6 ± 34.7 cells (range 25–1383) in the adult diet-manipulation experiment and 311.3 ± 4.2 cells (range 74–1046) in the developmental diet experiment. The total number of cells that were counted in the samples is accounted for in the analysis (see below). Of a total of 116 males from both experiments, the viability subsamples of 10 males had insufficient sperm, reducing the sample size for sperm viability to 106 males (adult diet experiment, high *n* = 30; restricted *n* = 29; developmental diet experiment, high *n* = 23, restricted *n* = 24).

For sperm morphology measurements, images of sperm cells were captured from each male’s ejaculate (×400 magnification; UB 200i Series Microscope and C13-ON camera, PROiSER R + D, Paterna, Spain). Pictures were analyzed in ImageJ to measure length of head, midpiece, and flagellum (mean number of sperm cells analyzed per male in the adult diet experiment = 18.6 ± 0.5 SE; range: 4–20; in the developmental diet experiment = 18.5 ± 0.3 SE; range: 13–20). The three measures of sperm morphology were condensed using a PCA, which returned one PC (henceforth sperm morphology PC1) with standard deviation ≥ 1, which accounted for 42% of the total variance and were used for further analysis (Supplementary Table 3). Sperm morphology PC1 was loaded negatively by flagellum length and positively by head and midpiece length (Supplementary Table 3). Of the total of 116 males from both experiments, the sperm morphology subsamples of 10 males had insufficient sperm, reducing the final sample size for sperm morphology to 107 males (adult diet experiment, high *n* = 30; restricted *n* = 30; developmental diet experiment, high *n* = 23, restricted *n* = 24).

After sperm assays, the male was dissected and the testes were removed under a dissection microscope (Leica S9i; Leica Microsystems Ltd., Heerbrugg, Switzerland) and weighed (Mettler Toledo XS105 scale). We removed one male (adult high-diet treatment) from the analyses as his testes were not properly developed which reduced the sample size to 115 males (adult diet experiment, high *n* = 29; restricted *n* = 30; developmental diet experiment, high *n* = 20, restricted *n* = 26).

### Statistical analyses

To account for block effects, that can emerge from sequential sampling, and to allow for statistical comparisons between Experiment 1 and 2, all traits, apart from the response variables in the female mate choice analysis, were converted to standardized trait values prior to analyses by dividing each trait value by the mean of the block for that trait. Since we were explicitly interested in the effects of both diet manipulation treatments (i.e., high vs. restricted diet) and when the diet experiments were applied (i.e., adult vs. juvenile), all models initially included diet treatment, experiment, and their interaction as fixed factors (unless otherwise stated).

First, we validated that male body condition was influenced by the diet treatment. To do this, we use a linear model with male condition (Fulton’s K) as the response variable and the diet treatment, the experiment (adult or developmental diet), and their interaction were entered as fixed factors. In the adult diet experiment, data on male condition were also available at the start of the diet treatment. Therefore, we compared adult condition (response variable) before and after the diet treatment in a linear mixed model with diet treatment, timepoint (at the beginning or end of the diet treatment), and their interaction, as fixed factors, and male ID as random factor.

Next, we examined if diet treatment and timing of diet manipulation (adult or developmental diet) influenced the expression of putative pre-copulatory sexually selected traits (beak length and coloration area) as well as male courtship (total count and duration of sexual behavior indices) and competitive behaviors (total count and duration of competitive behaviors). These (apart from competition behavior) were analyzed using linear models that included the trait as a response variable and diet treatment, experiment, and their interaction as fixed factors. The interaction was removed if non-significant. Competition behavior was analyzed using a linear mixed model to additionally include recording ID as random factor since the analysis is done per individual and not per recording. Body length was added as a covariate to models examining beak length to account for allometric effects. In models with coloration (coloration PC1 and PC2), body length and beak length were added as covariates. In models assessing male courtship behaviors, the total number of courtship behaviors were log10-transformed and the total duration of courtship behaviors was square-root transformed to improve the fit to a normal distribution.

Next, we examined whether females preferentially associate with the male of one treatment over the other using a strength of preference (SOP) score for the left male (arbitrarily defined without regard to diet treatment) as the time a female spends in the association zone of the left male divided by the total amount of time the female spend in the left and right association zones. SOP values range from 0 (females only spend time in the association zone of the male on the right) to 1 (females only spend time in the association zone of the male on the left). We then fit a linear model with SOP (left male) as the response variable, with diet treatment and experiment as fixed factors. To examine if male morphological traits influenced female preferences, we first calculate the difference in morphological trait values between the left and right male regarding body length, beak length, red coloration on the fins, red on beak, black on beak, yellow coloration on the fins, and black coloration on the fins. A linear model was fitted with SOP for the left male as the response variable and the differences in trait values together with experiment as fixed factors. The results did not change when we fitted each trait in separate models.

Finally, we examined the effect of diet restriction on sperm velocity, sperm viability, sperm morphology, and testes mass. For sperm viability, we calculated the proportion of live sperm in the samples from each male. Post-copulatory traits were assessed using linear models that included the trait as response variable and diet treatment, experiment, and their interaction, as fixed factors. Body length was added as a covariate to models examining testes mass to account for allometric effects. The model with sperm viability was weighted by the total number of cells counted (i.e., sum of live and dead cells per sample).

All analyses were performed in R 4.0.2 (R Core Team 2020) using the *lm* function from the *stats* package for linear models. The *lmer* and *glmer* functions from lme4 package (Bates et al. 2015) were used for the mixed models. The *Anova* function from the *car* package was used to obtain significance effects (Fox and Weisberg 2011). Model fit was assessed using model diagnostics and residual plots from base R. The *emmeans* package (Lenth 2019) was used to perform post hoc Tukey tests to identify differences in main effects. In all cases, non-significant interaction terms were removed from models and simplified models are presented.

## RESULTS

### Validating the efficacy of the diet treatments

**Figure 1:**
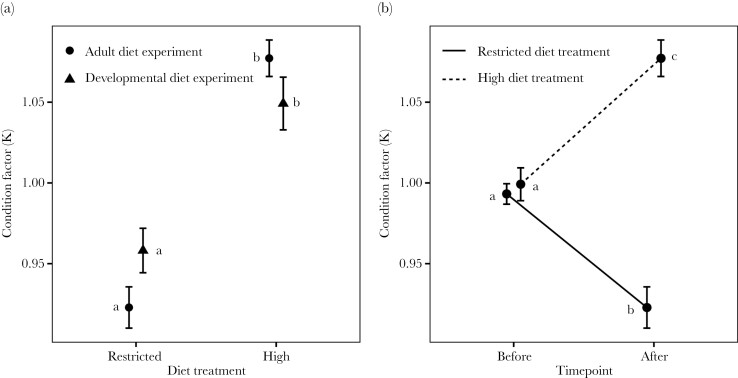
Validating the effects of diet manipulations on male condition (mean ± SE) standardized per block. The lowercase letters (a, b, c) in the figure represent the groupings from the post hoc tests. A) Condition factor after diet treatment for males in high and restricted diet treatment in adult and juvenile diet experiments (circle = adult diet experiment, triangle = juvenile diet experiment). B) Condition in the adult diet experiment for high treatment (dashed line) and restricted treatment (filled line) before and after diet treatment.

There was a significant interaction between diet treatment and experiment in our measure of male condition, Fulton’s K (Figure 1A; Table 1A). Yet despite this significant interaction term, post hoc tests revealed that males in the high diet treatment were in better condition at the end of the experiment than males in the restricted diet treatment in both the adult diet and developmental diet experiments (Figure 1A).

**Table 1:** The effects of diet treatment, experiment, and covariates (predictors) on male traits (standardized per block). A) Validating the effect of the diet manipulation on condition, B) effect on pre-copulatory traits from diet treatment, and C) effect on post-copulatory traits from diet treatment. The sample size in each statistical model is presented (*n*). Non-significant interaction effects were removed from the final models. Significant effects are shown in bold

Male trait	*n*	Predictors	*F*	*P*
**A. Condition**				
Condition factor (K)	116	**Treatment**	**91.13**	**<0.001**
		Experiment	0.14	0.71
		Treatment * Experiment	**5.54**	**0.02**
**B. Pre-copulatory traits**				
Beak length	98	**Treatment**	**13.34**	**<0.001**
		Experiment	0.21	0.64
		**Body length**	**22.98**	**<0.001**
Coloration PC1 (yellow on fins and black on beak)	98	Treatment	0.13	0.72
		Experiment	0.02	0.88
		Body length	3.56	0.06
		**Beak length**	**9.52**	**0.003**
Coloration PC2 (red on beak and fins)	98	**Treatment**	**7.42**	**0.008**
		Experiment	0.61	0.44
		Body length	1.51	0.22
		Beak length	0.85	0.36
Total number of sexual behaviors index	106	**Treatment**	**6.59**	**0.01**
		Experiment	0.01	0.94
Total duration of sexual behaviors index	106	Treatment	0.57	0.45
		Experiment	0.28	0.60
Total number of competitive behaviors^a^	100	Treatment	0.36	0.55
		Experiment	0.00	0.95
Total duration of competitive behaviors^a^	100	Treatment	0.10	0.75
		Experiment	0.15	0.70
**C. Post-copulatory traits**				
Sperm velocity (velocity PC1)	93	Treatment	2.60	0.11
		Experiment	0.34	0.56
Sperm viability	103	Treatment	1.20	0.27
		Experiment	2.74	0.10
Sperm morphology PC1	107	Treatment	0.09	0.76
		Experiment	0.00	0.99
Testes mass	115	**Treatment**	**29.38**	**<0.001**
		Experiment	0.01	0.93
		Treatment * Experiment	**6.39**	**0.01**
		**Body length**	**51.77**	**<0.001**

Competitive behaviors were analyzed using linear mixed model, that is, with a random factor of trial ID, which means the test statistic is Chi-square, not F.

For the adult diet experiment, we were able to assess changes in male condition factor before and after the diet manipulation. We detected a significant interaction between the time when condition was assessed and the diet treatment (Time effect, *Χ*^*2*^ = 0.21, df = 1, *P* = 0.64, Treatment effect, *Χ*^*2*^ = 43.21, df = 1, *P* < 0.001, Time * Treatment effect, *Χ*^*2*^ = 79.83, df = 1, *P* < 0.0001; Figure 1B). Indeed, while male condition did not differ between the diet treatment groups at the start of the experiment, by the end of the diet manipulation male condition factor increased in the adult high diet treatment and decreased in the adult restricted diet treatment (Figure 1B).

### Male pre-copulatory traits

**Figure 2:**
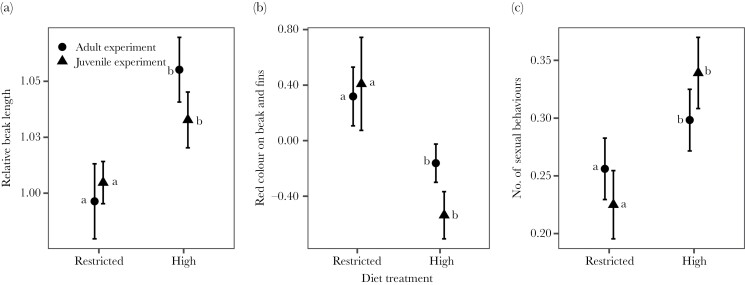
Effect of diet treatment on male pre-copulatory traits (mean ± SE) standardized per block. Note that there was no difference between the two experiments, only between the two diet treatments. The lower-case letters (a, b) represent the groupings from the post hoc tests, that is, showing that the traits did not differ between experiments, only between the two diet treatments. The adult (circle) and juvenile diet experiment (triangle) are presented separately. A) Beak length (corrected for body size) for the males in the high and restricted treatments in adult and juvenile diet experiment, B) area of red coloration on the beak (coloration PC2) for the males in the high and restricted treatments in adult and juvenile diet experiment, C) Total number of sexual behaviors index for the males in the high and restricted treatments in adult and juvenile diet experiment.

Males in the high diet treatment group had longer beaks after the diet treatment than males from the restricted diet (Figure 2A; Table 1B). These diet-mediated differences in beak length were consistent between the two diet experiments (Figure 2A; Table 1B). Coloration PC1, which describes variation in yellow on fins and black on beak, was not influenced by treatment or experiment (Table 1B). In contrast, coloration PC2, which describes the amount of red on beak and fins, was influenced by diet treatment (Figure 2B), with males from the restricted diet treatment group displaying larger area of red color on the beak and fins than males in the high diet treatment group (Table 1B). Coloration PC2 did not differ between the adult and developmental experiments (Table 1B).

In both the adult and developmental experiments, males performed a greater total number of sexual behaviors in the high diet treatment than in the restricted diet treatment (Figure 2C; Table 1B). There was no difference in the total duration of sexual behaviors between the diet treatments or experiments (Table 1B).

Interactions between males from the high and low diet treatments were frequent across competition trials. Displacements occurred in 82% of trials (46 of 56 trials). Across all trials, 3.82 ± 0.26 (mean ± SE) displacements occurred per trial. Lunges occurred in 50% of trials (28 of 56 trials) and 4.11 ± 0.35 (mean ± SE) lunges occurred per trial. Beak-locking was less common and occurred in only 23% of trials (13 of 56 trials). However, there was no difference in the number of competitive behaviors performed by males in either diet treatment or between the experiments (Table 1B). Similarly, swimming behind or following another male occurred in 96% of trials (54 of 56 trials) but were not related to diet treatment or experiment (Table 1B).

### Female preference in relation to male morphology

Male diet treatment did not influence who the females spent more time with (*F* = 0.1020, df = 1, *p* = 0.32), and this was consistent between the two experiments (*F* = 0.02, df = 1, *P* = 0.90). Female preference was also not influenced by the difference in male body length (*F* = 0.23, df = 1, *P* = 0.63), beak length (*F* = 0.90, df = 1, *P* = 0.35), red coloration on the fins (*F* = 0.59, df = 1, *P* = 0.45), red on beak (*F* = 0.00, df = 1, *P* = 0.97), black on beak (*F* = 2.18, df = 1, *P* = 0.15), yellow coloration on the fins (*F* = 0.26, df = 1, *P* = 0.61), or black coloration on the fins (*F* = 0.15, df = 1, *P* = 0.70), and this did not differ between experiments (*F* = 0.00, df = 1, *P* = 0.99).

### Male post-copulatory traits

**Figure 3:**
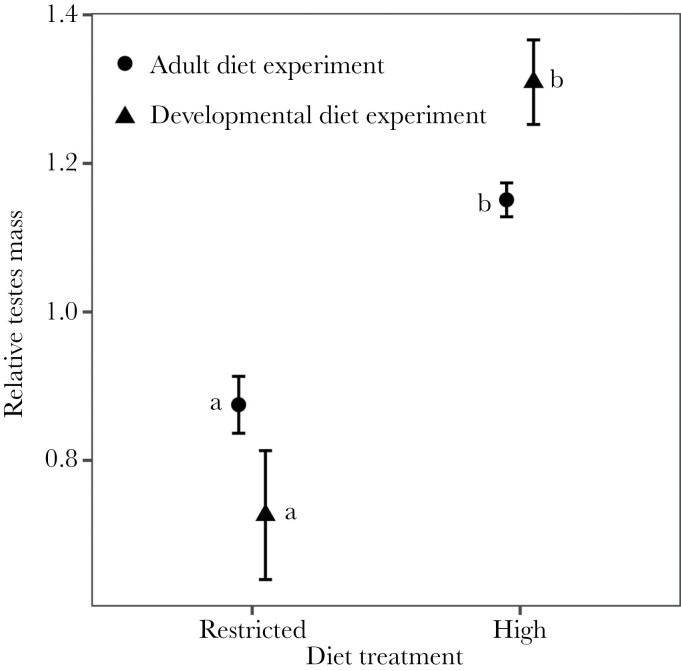
Effect of diet treatment on the testes mass (mean ± SE) as proportion of body mass (standardized per block) between males in the high and restricted treatments in the adult (circle) and juvenile diet experiment (triangle). Post hoc tests showed that males in the restricted diet treatment (a) differed from the males in the high diet treatment (b), but not between experiments.

Body-size corrected testis mass was significantly affected by diet treatment, and an interaction with experiment was also detected (Figure 3; Table 1C). Post hoc comparisons revealed that males maintained on a high-diet had larger testes than males maintained on a restricted-diet in both the adult and juvenile diet manipulation experiments (Figure 3; Table 1C). Males in the high-diet treatment group had testes making up on average ~3.5% of their total body mass, while testes mass of the males in the restricted-diet treatment group made up on average ~2.3% of their total body mass.

Neither diet treatment nor experimental treatment influenced sperm velocity or sperm morphology (Table 1C). Sperm viability was influenced by diet treatment (*F* = 5.08, df = 1, *P* = 0.02), experimental treatment (*F* = 20.08, df = 1, *P* < 0.001), and their interaction (*F* = 4.95, df = 1, *P* = 0.03) in the full dataset. However, these sperm viability effects were driven by three outliers. When these outliers were removed from the model, sperm viability was not influenced by diet treatment (Table 1C), although there remained a statistical trend suggesting that experiment influenced sperm viability, with sperm viability being higher in the males from the adult diet experiment compared with males from the developmental diet experiment (Table 1C).

## DISCUSSION

This study demonstrates that resource restriction during development and after reaching sexual maturity influence the expression of pre- and post-copulatory sexual traits in male halfbeaks. Males in the restricted diet treatments showed reductions in body condition, the total number of courtship behaviors they performed, the length of their beaks, and in their testicular investment compared with male in the high diet treatments. However, not all the sexual traits we examined exhibited resource-dependence, despite the less conservative approach used in this study, where fish were maintained on the diets throughout assays (rather than allowing males from restricted diet to return to control (ad libitum diet) conditions for assaying). Specifically, the duration of courtship behaviors, the amount of yellow coloration on fins and black coloration on beak, and all sperm quality traits were not affected by diet manipulations. Contrary to expectations, the area of red coloration on the beak and fins was lower in males maintained on the high compared with the restricted diet. Moreover, the pattern of resource-dependent expression of sexual traits was remarkably consistent between long-term diet restriction during development and short-term diet restriction after males reached sexual maturity, despite slight differences in experimental setup (social experience, type of food, age at assay) – which was also contrary to our expectations. Overall, these findings highlight the complexities of understanding resource dependent expression of sexual traits.

Our findings suggest that resource-dependent expression of sexual traits in male halfbeaks may influence their competitive ability. Most of the sexual traits that exhibit resource dependence in male halfbeaks have the potential to play a role in male–male competition. For example, male body condition and beak length are negatively influenced by resource limitations and these traits are likely to influence the outcome of competitive interactions. Males in better condition are often socially dominant and more likely to win male–male contests for access to mates or territories (e.g., Rowland 1989; Bisazza et al. 1996; Jennions and Backwell 1996; Barber 2002; Poulos and McCormick 2015). Thus, reductions in body condition after resource limitations could make male halfbeaks less competitive in male contests. Similarly, weapons used in male–male competition can also predict the outcome of aggressive dominance interactions (e.g., Jennions and Backwell 1996; Judge and Bonanno 2008). As male halfbeaks lock their beaks together during “wrestling” matches for social dominance, the reduction in beak size with resource restrictions could have an impact on male success during male contest competition, either by directly affecting a male’s ability to win or indirectly through threat displays prior to wrestling matches. Yet surprisingly, competitive behaviors did not differ between males fed high and restricted diets. In this study, males in a competition trial were similar in body size, which may have removed a competitive advantage from one male over the rival. In other taxa, larger body size offers an advantage in competitive interactions (e.g., Alcock 1995; Hagelin 2002; Benson & Basolo 2006; Liu et al. 2021). Size-matching of males may also have reduced within-pair differences in beak size, which may have an important role in competition. On the other hand, it is also possible that males were not motivated to compete since there was no female or other resource present to compete over. However, a recent study found that agonistic interactions between two males were highest in the absence of a female (Reuland et al. 2021). Clearly, the determinant(s) of success in competitive interactions in halfbeaks requires further study.

We also detected patterns of resource-dependent expression of post-copulatory sexual traits that could influence the outcome of sperm competition in halfbeaks. Specifically, males experiencing resource limitations invested less in testes mass (controlling for body size). Testis size is correlated with sperm production rates and number of sperm cells produced (Moller 1988, 1989) and a common proxy of sperm competition risk (Simmons and Fitzpatrick 2012). Sperm number is the primary determinant of male competitive success during sperm competition in multiple taxa (Simmons and Fitzpatrick 2012). Consequently, the lower investment in testicular tissue in males in the restricted diet treatments suggests that resource limitation may reduce male competitiveness during sperm competition in halfbeaks. However, whether sperm number influences male success during post-copulatory intra-sexual competition is not currently known for halfbeaks, although this is an avenue in need of exploration.

Resource restriction also impacted male courtship behaviors as male circling behaviors and mating attempts exhibited resource-dependent expression. These behaviors are a key part of male courtship and likely influence mate assessment and mating success (Greven 2010). While a direct link between these behaviors and male fertility has yet to be established, mate assessment behaviors are linked with male fitness and paternity share in a wide range of species (Evans and Magurran 2001; Schneider and Lesmono 2009; Chargé et al. 2010; Gao et al. 2019). The effect of mating rate on fertilization success or fitness seems more complex, with both positive and negative correlations reported (Warner et al. 1995; Tregenza and Wedell 1998; Jones and Elgar 2004; Pérez-Staples et al. 2010; Pischedda and Rice 2011), possibly interacting with the number of sperm transferred during matings. Therefore, by performing less circling and mating attempts, while simultaneously having smaller total sperm reserves, males in the restricted diet treatments will likely have reduced fertilization success compared with males in the high diet treatments. Reductions in courtship behaviors are common after resource restriction in many species. For example, mating rates in mosquitofish (Fox et al. 2019) and bedbugs (Kaldun and Otti 2016), the number of sigmoid displays in male guppies (Devigili et al. 2013; Rahman et al. 2013, 2014; Evans et al. 2015; Cattelan et al. 2019), the calling rate and effort of field crickets (Scheuber et al. 2003) and frogs (Wang et al. 2018), and the gift handling and number of pulses by spiders (Eberhard et al. 2020) are all reduced after resource restriction. Yet not all halfbeak courtship behaviors were affected by resource restriction. Courtship behaviors of swimming under, nipping, and checking, were not affected by diet restriction, suggesting they may be less costly to perform.

An important finding from this study is the general consistency in the resource dependent response in sexual traits between the adult and juvenile diet manipulation experiments. Broadly speaking, this indicates that a relatively short diet restriction of one month during sexual maturity had a similar impact on sexual traits as a prolonged diet restriction of around 4 months until sexual maturation. In animals with indeterminate growth (e.g., fish, mollusks, and some reptiles), sexually selected traits may be sensitive to resource availability throughout their lifetime, even after sexual maturity (Heino and Kaitala 1999). Indeed, the consistency between the two experiments in this study suggests that the period of development after reaching sexual maturity is a more critical timepoint in terms of investment in sexually selected traits. A recent meta-analysis showed that ejaculate traits in fish are generally more sensitive to adult compared with juvenile resource limitation (Macartney et al. 2019). Yet in halfbeaks, the only post-copulatory trait that showed resource-dependent expression (i.e., testes mass) was similarly affected in the two experiments. Despite this apparent contradiction with the general pattern observed in fishes, it is noteworthy that of the studies of fish summarized in Macartney et al. (2019)’s meta-analysis, resource restriction during development have only been examined in two species: mosquitofish (*G. holbrooki*; Vega-Trejo et al. 2016) and sticklebacks (*Gasterostaus aculeatus*; Mehlis et al. 2015). Moreover, resource restriction during development and at sexual maturity in mosquitofish both led to a reduction in sperm number and sperm replenishment (O’Dea et al. 2014; Vega-Trejo et al. 2016), which broadly matches the reductions in testes mass after resource restriction in both experiments we observed in halfbeaks.

Resource-dependence in sexual traits appear less linked with mate choice in halfbeaks. For example, females were not influenced by the male diet treatment in their mate choice nor by differences between the males in their morphological traits. Since the males in the dichotomous trials were paired with no regard to their coloration, it may be that on average, there was low within-pair difference in morphology, making it difficult for the female to choose between the two males (McNeil et al. 2021). On the other hand, females are most likely to encounter random variation and subtle differences between males in natural populations. Notably, the females spent in general slightly more time with one male in the trials (on average 65% vs. 35%), which suggests we did not capture what the females’ paid attention to in this experiment. For example, it would be interesting to link variation in male courtship behavior (which showed resource dependence) with female preference in the halfbeak.

In many species, including halfbeaks (Reuland et al. 2021; McNeil et al. 2021), females base mate choice decisions on the conspicuous colors displayed by males (e.g., Hill 1990; Houde 1997; Perez-Rodriguez and Vinuela 2008). Yet in this study, the majority of coloration traits either weren’t influenced by resource restriction or were expressed *more* in the restricted-diet treatment in the case of red coloration on the beak and fins. The lack of reduction in coloration after diet restriction was unexpected, as the size and intensity of carotenoid-based coloration (e.g., red/orange coloration) is condition-dependent in many species (e.g., Naguib and Nemitz 2007; Perez-Rodriguez and Vinuela 2008; Devigili et al. 2013; Jiménez-Cortés and Córdoba-Aguilar 2013) and can change rapidly in response to resource restriction (e.g., Devigili et al. 2013). There are several potential explanations for these findings. First, sexual ornaments in halfbeaks may not be dependent on ingested nutrients through the diet. However, a more likely explanation for our results is that small amounts of carotenoids in the diet are required to grow and express sexual coloration traits in halfbeaks, and males received sufficient amounts in the restricted diet treatment. Experimental manipulation of carotenoid levels would be a good way to test this hypothesis. A third possible explanation is that color intensity is resource-dependent, whereas the total area of color on the body is not, which is an interesting prospect for future investigation. Interestingly, restricted diet males had larger area of red coloration on the beak and fins after diet treatment in both experiments. Three-spined sticklebacks also show an increase in red coloration after diet restriction, which was hypothesized to maximize chances of mating through female mate choice (Mehlis et al. 2015). However, we found that female halfbeaks did not show a preference based on differences in red beak or fin coloration. Additionally, previous studies on halfbeak female mate choice generally show avoidance of red males (Reuland et al. 2020; McNeil et al. 2021). It therefore seems that a strategy to maximize mating opportunities through increasing red coloration would be ineffective. It may be that males from the high diet group suppress their red coloration. To nail down whether the difference in red coloration between the treatment groups stem from an increase in the restricted diet treatment or decrease in the high diet treatment, it would be interesting to monitor the change in red coloration over the course of a period of resource manipulation. Furthermore, if the beak is more important in male–male competition rather than female mate choice, the red coloration may be used to signal rival males. We do not yet know the role of the beak or its coloration in competitive interactions. The role of male–male competition and this potential weapon in the halfbeak is an intriguing avenue for future research which will aid in disentangling the contributing effects of intra- and inter-sexual selection.

None of the sperm quality traits investigated in this study were resource-dependent. Overall, ejaculate traits in fish are less sensitive to available nutrients than in mammals and insects (Macartney et al. 2019). However, multiple studies have demonstrated the costliness of ejaculates in various taxa (Preston et al. 2001; Kahrl and Cox 2015), including fish (Nakatsuru and Kramer 1982; Gasparini et al. 2013). In this study, the velocity, viability, and morphology of sperm cells were not significantly affected by diet treatment. Interestingly, dominance hierarchy influences halfbeak sperm velocity and viability, but only when encounters with a rival are frequent (Reuland et al. 2021). Since dominant males were also in better condition than subordinate counterparts, this may suggest that condition dependence in halfbeak ejaculate traits is mediated through differences in dominance rank and/or the level of aggressive interactions (Reuland et al. 2021). This provides further evidence that dominance and intra-sexual selection should be incorporated in studies examining the variation in sexually selected traits. However, another explanation for the observed lack of resource dependence in ejaculate traits may be that sperm qualities such as viability and velocity are so fundamental to fertilization success that they are buffered against environmental impact via past selection (adaptive canalization hypothesis; Gibson and Wagner 2000), such that other, less important traits can be impacted by resource restriction, while fertilization capacity (and by extension, reproductive fitness) remains intact (Macartney et al. 2019). Future work should therefore focus on the relative importance of sperm number, velocity, viability, and morphology in fertilization success.

Our study demonstrates that certain sexually selected traits are costly to produce and maintain. Overall, we argue that the fitness implications of resource-dependent expression of sexual traits in male halfbeaks may primarily be linked to reductions in mating opportunities and reduced success during intra-sexual (male–male) competition. An individual more capable of acquiring resources may display quality to potential mates through elaborate sexual behaviors and have higher fertilization success through larger sperm production capacities. The study also highlights the importance of incorporating many traits, as well as many aspects of the same trait (such as behavior), and a careful consideration of the timing of the resource manipulation in studies of resource dependence. Additionally, since sexual selection is a process that requires the influence of both sexes, future research should also investigate the resource dependence of traits in females, such as mate preferences and choosiness (Cotton et al. 2006).

## Supplementary Material

arac060_suppl_Supplementary_Figure_S1

arac060_suppl_Supplementary_Material

arac060_suppl_Supplementary_Resource_1

arac060_suppl_Supplementary_Resource_2

arac060_suppl_Supplementary_Resource_3

arac060_suppl_Supplementary_Resource_4
