# Supplementary figures and images for: Resource-dependent investment in male sexual traits in a viviparous fish
Source: Behav Ecol. 2022 Jun 23;33(5):954–66. doi: 10.1093/beheco/arac060 (PMC9639585; doi:10.1093/beheco/arac060)

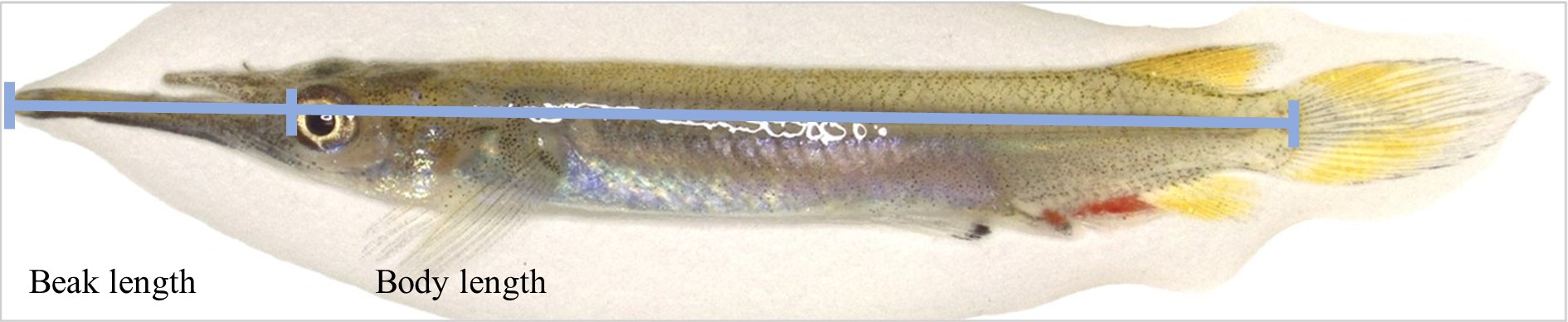

Supplement: arac060_suppl_Supplementary_Figure_S1 [file arac060_suppl_supplementary_figure_s1.jpeg]
