# Supplementary material for: Resource-dependent investment in male sexual traits in a viviparous fish
Source: Behav Ecol. 2022 Jun 23;33(5):954–66. doi: 10.1093/beheco/arac060 (PMC9639585; doi:10.1093/beheco/arac060)
Supplement: arac060_suppl_Supplementary_Material [file arac060_suppl_supplementary_material.docx]

Supplementary material for

Resource-dependent investment in male sexual traits in a viviparous fish

Figure legends

**Supplementary Figure 1.** A male halfbeak. Beak length was measured from the anterior tip of the beak to the anterior part of the eye, and body length was measured from the anterior part of the eye to the caudal peduncle.

Tables

**Supplementary Table 1**: Colouration PC1 and PC2 component loadings. Loading values that are within 70% of the largest loading value for each PC are considered to contribute to the PC (marked in bold). All colouration values were standardized within experimental blocks prior to their inclusion in the PCA. Only PCs with standard deviation ≥ 1 are presented; here PC1 and PC2. The proportion of variance and cumulative proportion of variance is presented for PC1 and PC2.

| Loading | Colouration PC1 | Colouration PC2 |
| --- | --- | --- |
| Total yellow on fins | **0.64** | 0.06 |
| Total red on fins | 0.17 | **0.52** |
| Black on dorsal fin | 0.37 | 0.32 |
| Red on beak | -0.09 | **0.74** |
| Black on beak | **0.65** | -0.27 |
|  |  |  |
| Standard deviation | 1.24 | 1.13 |
| Proportion of Variance | 0.31 | 0.25 |
| Cumulative Proportion of Variance | 0.31 | 0.56 |

**Supplementary Table 2**: Sperm velocity PC1 component loadings. Loading values that are within 70% of the largest loading value for each PC are considered to contribute to the PC (marked in bold). All sperm velocity values, including the average path velocity (VAP), straight-line velocity (VSL) and curvilinear velocity (VCL), were standardized within experimental blocks prior to their inclusion in the PCA. Only PCs with standard deviation ≥ 1 are presented; here PC1. The proportion of variance and cumulative proportion of variance is presented for PC1.

| Loading | Sperm velocity PC1 |
| --- | --- |
| VCL | **0.57** |
| VSL | **0.58** |
| VAP | **0.59** |
|  |  |
| Standard deviation | 1.70 |
| Proportion of Variance | 0.96 |
| Cumulative Proportion of Variance | 0.96 |

**Supplementary Table 3**: Sperm morphology PC1 component loadings. Loading values that are within 70% of the largest loading value for each PC are considered to contribute to the PC (marked in bold). All sperm morphology values, including sperm head, midpiece and flagellum length, were standardized within experimental blocks prior to their inclusion in the PCA. Only PCs with standard deviation ≥ 1 are presented; here PC1. The proportion of variance and cumulative proportion of variance is presented for PC1.

| Loading | Sperm morphology PC1 |
| --- | --- |
| Sperm head length | **0.45** |
| Sperm midpiece length | **0.63** |
| Sperm flagellum length | **-0.63** |
|  |  |
| Standard deviation | 1.12 |
| Proportion of Variance | 0.42 |
| Cumulative Proportion of Variance | 0.42 |
